# *Notes from the Field*: Public Health Response to a Human Immunodeficiency Virus Outbreak Associated with Unsafe Injection Practices — Roka Commune, Cambodia, 2016

**DOI:** 10.15585/mmwr.mm6704a6

**Published:** 2018-02-02

**Authors:** Ugonna C. Ijeoma, Sin Sansam, Sok Srun, Hoy Vannara, Sou Sanith, Tek Sopheap, Robert D. Newman, Renuka Gadde, Selenic Dejana, Ahmed Saadani Hassani, Vanthy Ly, Bakary Drammeh, Anindya De, Johnita Byrd, Naomi Bock

**Affiliations:** ^1^Epidemic Intelligence Service CDC; ^2^HIV Prevention Branch, Division of Global Health and TB, CDC; ^3^Ministry of Health, Phnom Penh, Cambodia; ^4^Becton, Dickinson and Company, Franklin Lakes, New Jersey.

Cambodians receive 0.8–5.9 therapeutic injections per person per year, one of the highest reported rates worldwide (*1*,*2*). Appropriate medical injections and infusions can be health sustaining or lifesaving; however, improper administration can have detrimental health consequences, including infectious disease transmission (*3*). In 2000, it was estimated that worldwide, unsafe injection and waste disposal practices account for 260,000 new human immunodeficiency virus (HIV) infections annually (*3*).

A case-control study conducted as part of an investigation of an outbreak of 242 new cases of HIV infection among residents of Roka Commune, Battambang Province, Cambodia, from December 2014 through February 2015, (*4*) identified unsafe medical injection practices by an unlicensed health care practitioner as the likely source of the outbreak, highlighting the potential for unsafe therapeutic injection practices to contribute to HIV transmission in Cambodia. After this outbreak, the government of Cambodia implemented new regulations to prohibit unlicensed medical practices (*5*). Although the outbreak was associated with the unregulated health sector, it prompted an assessment of injection safety practices among licensed health care workers in Cambodia, given the high public demand for medical injections. To identify potential gaps in safe injection practices, the Cambodia Ministry of Health (MOH) partnered with CDC and the medical technology company Becton Dickinson (Franklin Lakes, New Jersey) to conduct a rapid assessment of injection practices at public health facilities.

From September 26–29, 2016, a team of medical officers from CDC and a clinical team from Becton Dickinson with expertise in infection control assisted the Cambodia MOH in implementation of the rapid assessment. A cross-sectional study* was conducted among the 15 main government health care facilities in Battambang and Pursat provinces, which are among the provinces with the highest medical injection rates (*1*) and are in close proximity to the site of the 2014–2015 HIV outbreak. A World Health Organization (WHO) standardized injection practices assessment tool (*6*) was used to interview licensed health care workers, including physicians, nurses, and laboratory technicians, and observe all injections administered. Injection technique was evaluated using a standardized checklist. The interview questions ascertained knowledge, attitudes, and practices regarding injection use and safety. Frequencies were calculated, and, given the limited sample size, exact 95% confidence intervals were estimated using statistical software.

A total of 115 injection events were observed, and 39 health care workers were interviewed (Table); 99% of injections were administered with needles and syringes taken from unopened, sterile packs. However, patient identification was not confirmed before injection in 54% of events, hand hygiene procedures did not precede injection in 79% of events, and a new cotton swab was used in only 36% of events. Observation of safety practices demonstrated that 63% of health care workers recapped needles after use; 51% were recapped with two hands. Less than half (48%) of sharps containers were appropriately placed within arm’s length of health care worker; however, most needles (83%) were still placed in a sharps container immediately after use. All 39 interviewed health care workers knew that HIV could be transmitted through unsafe injection practices, but fewer were aware of the potential for transmission of hepatitis B virus (79%) and hepatitis C virus (62%) through this route. Finally, 28% of health care workers reported ever experiencing a needle stick injury, and 49% reported ever receiving formal injection safety training.

**TABLE:** Assessment of safe injection knowledge, attitudes, and practices among health care workers — Battambang and Pursat provinces, Cambodia, 2016

Assessment component (No. assessed)	No.	% (95% CI)
**Observation of injection administration (115)**		
**Procedures affecting patient safety**		
Sterile needle/syringe used for injection (112)	111	99 (95–100)
Patient identification not confirmed before injection (113)	61	54 (44–63)
Hand hygiene not performed before injection administration (115)	91	79 (71–86)
Injection site cleaned with a newly moistened cotton swab (110)	40	36 (27–46)
**Procedures affecting health care worker safety**		
Needle recapped after use (104)	65	63 (52–72)
Recapped with two hands (65)	33	51 (38–63)
Sharps container placed within arm’s reach of health care worker (106)	51	48 (38–58)
Needle disposed of in sharps container immediately after use (109)	91	83 (75–90)
**Health care worker interviews (39)**		
**Aware of disease transmission via unsafe injections**		
Human immunodeficiency virus	39	100 (91–100)
Hepatitis B	31	79 (64–91)
Hepatitis C	24	62 (45–77)
Ever experienced needle-stick injury	11	28 (15–45)
Received formal training on injection safety practices	19	49 (32–65)

**Abbreviation:** CI = confidence interval.

Although this study found little reuse of injection equipment and high knowledge of the risks of unsafe injection practices related to HIV transmission, none of the observed injections fully adhered to WHO standards of practice, thus potentially compromising both patient and health care worker safety. To address these gaps, an intensive training curriculum on safe injection practices for health care workers is being developed by Becton Dickinson with technical support from CDC. After review and approval by the Cambodia MOH, expert master trainers will administer this training in the same health care facilities where the baseline assessment was conducted. The impact of the training on the improvement of injection and phlebotomy practices will be measured with a follow-up assessment in the same facilities where the baseline assessment was conducted. Health assessment findings are also contributing to the revision of current policies, information education and communication resources, and the development of job aids on safe injection practices.

## Footnotes

* This study was conducted with the approval of the Cambodia National Ethics Committee for Health Research.
